# Optimal viscosity modelling of 10W40 oil-based MWCNT (40%)-TiO_2_ (60%) nanofluid using Response Surface Methodology (RSM)

**DOI:** 10.1016/j.heliyon.2022.e11944

**Published:** 2022-11-28

**Authors:** Mohammad Hemmat Esfe, Sayyid Majid Motallebi, Davood Toghraie

**Affiliations:** a Department of Mechanical Engineering, Imam Hossein University, Tehran, Iran; b Department of Mechanical Engineering, Khomeinishahr Branch, Islamic Azad University, Khomeinishahr, Iran

**Keywords:** Optimal modeling, Dynamic viscosity, Hybrid nanofluids, RSM, Thermophysical property

## Abstract

The science of nanofluids is still fairly new and due to this, the properties of many nanofluids are yet to be explored. Therefore, equations for precise calculations in this field are not available yet. For this reason, as a thermophysical property of an MWCNT (40%)/TiO_2_ (60%) hybrid nanofluid (HNF), in this research, the viscosity of HNF with 10W40 oil as the base fluid, in a temperature range of T = 5–55 °C and with solid volume fractions of SVF = 0.5–1% is studied and modelled. The viscosity of the nanofluid was examined in different conditions. Lab data were used to model dynamic viscosity of HNF using the Response Surface Methodology (RSM), and first, second, third, fourth and fifth-order models were created. An analysis of the statistical parameters concluded that with a correlation coefficient of 0.9999, the fifth-order model is the best performer. The trend of alterations in viscosity shows that an increase in temperature has great effects on viscosity, and its influence is also more important than that of changes in shear rate (SR) and SVF. Optimal viscosity was also calculated and was equal to 158.1 mPa.sec at SVF = 0.05 %, SR = 11,997 s^−^^1^ and T = 14.97 °C.

## Teaser

Optimal modeling; Dynamic viscosity; Hybrid nanofluids; Response surface methodology (RSM).

## 1 Introduction

Undoubtedly, in the last decade, nanotechnology and its emergence have caused many advances in engineering sciences, and various researchers have conducted a lot of research on different aspects of nanoscience, including different areas of particles, powders, fibers, fluids, etc. [1, 2, 3, 4, 5]. In addition to this, lubrication and the use of lubricants in various industrial processes to reduce friction is one of the challenges that encourages researchers to use high-performance lubricants; the issue of viscosity (µ) and its reduction with temperature (T) and its increase with particle concentration have always been a concern of researchers, and researchers are looking for a tool to increase the thermal properties of fluids and control their viscosity [6, 7]. Heat transfer and thermal issues are the most important phenomena in various industries [8, 9, 10]. Many instruments use conventional fluids such as water and ethylene glycol to heat transfer, but these fluids are not very efficient at this task. Multiple experiments were conducted to optimize fluids for this purpose. Hosseinzadeh et al. [11], investigated the types of fluids in the thermal conductivity of a closed two-phase thermosyphon. The fluids in question included water, water-hexanol, water-pentanol and water-butanol. 1-butanol with 6% concentration was the best performer as it has the maximum difference of surface tension with water. Besides the type of fluid, the effects of filling ratio, input power and flow rate of the cooler on the thermal and resistance performance were also reviewed in the article. Maxwell [12] introduced nanomaterials, a type of material in the nanometer scale of size, and Choi [13] added this material to fluids in hopes of improving the process of heat transfer using nano-fluids for the first time. Nanofluids are made of a nanoparticle such as ZnO, MWCNT, CeO_2_, Fe_3_O_4_ & CuO and a base fluid such as water [14, 15, 16, 17, 18]. In the research conducted by Mogharrebi et al. [19], the flow of a magnetohydrodynamic nanofluid containing oxytactic micro-organisms, over a rotating cone was examined. The governing equations were solved with the Runge–Kutta fifth-order method. In this work, the effect of various factors on speed, temperature and concentration is measured. The results of this study show that changing the magnetic parameter from 0 to 1 reduces the temperature distribution by about 3.11%. Jayaprakash et al. [20] studied the convective heat transfer efficiency and the Arrhenius activation energy of HNFs consisting of CoFe_2_O_4_ & TiO_2_ nanoparticles in water. A mathematical model was created that illustrated the radiative heat transfer over a curved stretching sheet with regards to injection/suction and Arrhenius activation energy. The results revealed that with rising values of radiation parameter and Biot number, the dimensionless temperature increases. Also stronger suction will lead to an increase in the dimensionless rate. Hemmat Esfe [21] modelled heat transfer and pressure drops using the relative Nusselt number, and examined the relative pressure drop of a water-based nanofluid containing silver nanoparticles, in a double tube heat exchanger. The Reynolds number and concentration were the input parameters of the neural network. Also, a neural network with tansig and logsig transfer functions was compared to another one with the RBF transfer function. The topology with the RBF transfer function outperformed other topologies. The Nusselt number and the relative pressure drop had regression coefficients of 99.76% and 99.54% respectively. Bhatti et al. [22] investigated the effect of diamond (C) and silica nanoparticles on a solar thermal collector with water base fluid. The goal of this research was to optimize the performance of the heat-transfer fluid in a solar thermal collector. Permeability, viscous dissipation function and magnetic field were also considered effective parameters. The nonlinear differential equations governing the problem were solved using the sequential linearization method. It was concluded from the results that the presence of mentioned nanoparticles significantly increases the velocity profile and improves the temperature profile. Hosseinzadeh et al. [23] investigated the effect of SiO2 superhydrophobic coating and a self-wetting fluid on thermal conductivity in a two-phase closed thermosyphon. The Super-hydrophobic coating was applied to the condenser and 1-Butanol was used as the self-rewetting fluid. The super-hydrophobic coating was created through synthesizing SiO_2_ nanoparticles and surface engineering. Results indicated that the super-hydrophobic coating caused a 13.34% increase in the convective heat transfer coefficient at a heat input of 250 W. Benos et al. [24] surveyed the effects of agglomeration in a water/CNT nanofluid on convective heat transfer in presence of a magnetic field and in hydrodynamic terms. A 2-dimensional rectangular Couette flow was the geometry in question. The results showed that rheological properties are highly influential on heat transfer and flow. Convection decreased after increasing the amount of CNT in the fluid, which lead to decreased flow rate and heat transfer. By studying various nanofluids, researchers came upon an understanding that these fluids can have noteworthy effects on certain properties of the base fluid, such as its viscosity and thermal conductivity, and therefore are capable of optimizing their heat transfer characteristics [25, 26, 27, 28]. Viscosity is a fluid’s resistance to stress and it is one of the important properties that affect heat transfer. Much effort has been made to optimize the viscosity of nanofluids. These researches include experimental and numerical surveys and simulations [29, 30, 31]. Asadi et al. [32], performed an experimental evaluation on the viscosity of MWCNT-MgO (20–80)/SAE50 HNF in multiple temperatures and SVFs. The T and concentration range in this work were respectively 25–50 °C and 2–25%. The results show an increase in viscosity with temperature and SVF. The maximum increment in viscosity for a fluid with SVF = 2% at T = 60 °C was 65%. Hemmat Esfe [33] studied the viscosity of HNFs consisting of CuO & MWCNT with a ratio of 85%–15% respectively, and 10W40 oil as the base to explore the applications of nanofluids in internal combustion engines. Experiments were conducted with SVF = 0.5–1%, SR = 2666.6–11,999.7 s^−1^ and in a T range between 5 and 55 °C. The maximum increment of viscosity was observed at SVF = 1% and was equal to 43.52%. To optimize the viscosity of an oil-based fullerene nanofluid, Ahmadi et al. [34], attempted an experimental study on such material. Nanofluids were prepared with a homogenizer using the two-step method. Nanoparticles were created in SVF = 0.05–2%. The results concluded that at the investigated temperatures the behaviour of the nanofluid was non-Newtonian. Also, the nanofluid had maximum viscosity at SVF = 0.4%. Numerical methods and simulations can save time and resources and are therefore important. Using ANNs to investigate viscosity was the focus of some researchers [35, 36, 37, 38]. Hemmat et al. [39] investigated the rheological behavior of the HNF containing MWCNT-SiO_2_ (10:90) with the response surface methodology (RSM). The main goal of this research was to present a new correlation. According to the results of the RSM, in two-variable relationships, the existence of the independent variable of temperature with volume fraction increases the accuracy of the mathematical relationship compared to the use of SR and SVF. Hemmat Esfe et al. [40] proposed a mathematical equation and a model for the viscosity of ZrO_2_-MWCNT HNF using the curve fitting method. Experimental data was obtained from Nanofluids with SVF = 0.05%, 0.1%, 0.25%, 0.5%, 0.75%, 0.1% and in T = 5–55 °C. Evidence of non-Newtonian and pseudoplastic behaviour from the nano-lubricant was visible in the results. The correlation coefficient of the viscosity equation was 0.9905. Khetib et al. [41] investigated the viscosity of CuO nanofluid based on paraffin with RSM. The data used for modelling was obtained from experiments in T = 25–100 °C and mass fractions of 0.25–6%. RSM shows that the obtained results from the third degree polynomial are more accurate than the second degree and linear polynomial. The cubic model had a maximum margin of deviation equal to 10.482% and R^2^ = 0.923. In this research, the analysis of the thermophysical property of viscosity for the MWCNT (40%)/TiO_2_ (60%)/10W40 HNF is done differently from previous attempts. Modelling the viscosity of the MWCNT (40%)/TiO_2_ (60%)/10W40 HNF based on effective parameters, and also, finding the optimal viscosity are the main goals of this study. To validate this model several statistical analyzes are presented. Plots were used to Investigate the nanofluid’s viscosity characteristics in different conditions. The influence of multiple parameters on the nanofluid’s viscosity was measured using variance and perturbation analysis.

## 2 Methodology

HNFs were created by preparing a mixture of MWCNT & TiO_2_ nanoparticles with a ratio of 40% and 60% respectively and adding it to 10W40 oil. Nanoparticles were weighed with an accuracy of 0.0001 g. A magnetic stirrer was used to stabilize the nanofluid for 2 hours, but the results were not satisfactory, therefore an ultrasonic stirrer was alternatively used for 1 h, which yielded adequate results and stabilized the nanofluid. To measure viscosity, a CAP2000+ Brookfield viscometer was used. The samples were measured twice and the average of the numbers was recorded. Data processing and interpretation were done via Response Surface Methodology (RSM). The recorded experimental data were used in modelling using the RSM and multiple models were created. The performance of each model will be evaluated and interpreted using statistical graphs and parameters, and the best-performing model will be determined.

### 2.1 RSM

With the help of different mathematical and statistical techniques, the RSM can be applied to different processes. The applications of this methodology include development, formulation, design and optimization of new products. In general, the Response Surface Methodology was first used by Box and Draper. In this methodology, we have one or more independent variables and one or more dependent variables or responses. The purpose of the methodology is to find how dependent variables change with respect to independent variables, in statistical terms. For example, in product development the response variable is y and the dependent variables or input variables are $\xi_{1}$, $\xi_{2}$, …, $\xi_{k}$. To create a model and present the mathematical relation, y should be written in terms of input variables. Therefore, it is converted into Eq. (1):

(1) $$y=f\left(\xi_{1},\xi_{2},\ldots ,\xi_{k}\right)+\varepsilon$$

In Eq. (1), ε is the term that describes variables that are not included in *f*, but can cause an error. ε includes measurement errors and a series of inherent issues of the system or the process. As the true form of the response function of (*f*) is unknown, it must be approximated (η). The deployment of RSM depends on the experimenter's ability to provide accurate raw data as they form the basis of calculations and statistical models. Polynomial functions are normally used to model processes. These functions are similar to first or second-order equations. In Eq. (2) a first-order model is presented.

(2) $$\eta =\beta_{0}+\beta_{1}x_{1}+\beta_{2}x_{2}$$

But a first order equation is generally similar to Eq. (3):

(3) $$\eta =\beta_{0}+\beta_{1}x_{1}+\beta_{2}x_{2}+\beta_{12}x_{1}x_{2}$$

But the second order model can be presented as Eq. (4):

(4) $$\eta =\beta_{0}+\sum_{i=1}^{n}\beta_{i}x_{i}+\sum_{i=1}^{n}\;\beta_{ii}{x_{ii}}^{2}+\sum_{i<j=1}^{n}\beta_{ij}x_{i}x_{j}$$

Higher order models are also used in the RSM. This method expresses equations such as the first and second-order equations presented above. RSM outputs are only appropriate and useable if they can precisely predict the response values with minimal error.

## 3 Results

### 3.1 Presenting models of different orders

Modelling lab data is one of the favourite tasks of many researchers. In this section different models are presented based on 173 experimental recordings, which are the viscosity of MWCNT (40%)-TiO_2_ (60%)/10W40 HNF in different conditions. Quadratic, Cubic, Quartic and Fifth order models are presented. Then, each one is further examined and the best model is selected. Eqs. (5), (6), (7), and (8) represent these models.

(5) $$\begin{array}{l} \mu =604.05051-21.76077T+113.31886\text{SVF}-0.021746\gamma \;-2.29394T*\varphi +0.000404T*\gamma +0.215263T² \end{array}$$

(6) $$\begin{array}{l} \mu =665.73129-36.78817T-5.00744T*\text{SVF}+0.001553T*\gamma \;+0.692247T²+340.08439\varphi ²-5.33855E-06\text{SR}²+0.067903\;+0.067903T²*\varphi -0.000041T²*\gamma -1.12684T*\varphi ²+1.35792E\;-07T*\gamma ²-1.17242E-07\text{SVF}*\gamma ²-0.003236T³-199.45771\varphi ³ \end{array}$$

(7) $$\begin{array}{l} \mu =679.50108-39.30384T+285.77180\varphi -0.029515\gamma \;-16.48578T\varphi +1.29479T²+0.363583T²\varphi +0.000025T²\gamma \;+2.87801T\varphi ²+3.75433E-09\varphi \gamma ²-0.024025T³-191.48492\varphi ³\;+2.41093E-10\gamma ³-0.027642T²\text{SVF}²+1.78833E-09T\varphi \gamma \;-0.002850T³\varphi -2.71540E-07T³\gamma -5.77977E-12T\gamma ³\;+0.000175T⁴+112.19094\varphi ⁴+4.22224E-15\gamma ⁴ \end{array}$$

(8) $$\begin{array}{l} \text{Sqrt}\left(\mu \right)=26.42373-0.851323T+25.02353\varphi -0.001563\gamma \;-0.190633T\varphi +8.71244E-06T\gamma +0.018899T²\;-123.87215\varphi ²+2.37042E-07\gamma ²+0.003716T²\varphi \;-0.000271T³+311.11499\varphi ³-1.61816E-11\gamma ³+2.51070E\;-11T\varphi \gamma ²-1.87448E-09\varphi ²\gamma ²-0.000030T³\varphi -2.81856E\;-14T\gamma ³+1.76385E-06T⁴-340.73524\varphi ⁴+2.44270E\;-10T³\varphi \gamma -1.61657E-11T⁴\gamma +0.014451T\varphi ⁴+133.29172\varphi^{5}\;+3.33301E-20\gamma^{5} \end{array}$$

### 3.2 Specifying the best model

#### 3.2.1 Evaluating accuracy-related statistical parameters of the models

##### 3.2.1.1 R-squared interpretation

To identify the best model, statistical parameters and graphs that depict the accuracy of the models were used. Adjusted R^2^, Predicted R^2^ and Std. Dev are the statistical parameters used in this study. The presented plots include residual plots, normal probability, Box-Cox plots and real vs predicted response values. To predict the accuracy of the proposed models, adjusted R-squared and predicted R-squared parameters are of higher priority than the R-squared parameter. By adding certain terms to the R-squared, it can be artificially modified into a model, even if these terms are not statistically significant. The closer a models R-squared value is to 1, the more accurate the model is. R-squared values for quadratic, cubic, quartic and fifth-order models are presented in Table 1 and are respectively equal to 0.9937, 0.9993, 0.9997 & 0.9999. As it can be seen, R^2^ is close to 1 for all of the models, but the fifth-order model has higher accuracy.

**Table 1:** R-squared for different models examined.

Source	Quadratic	Cubic	Quartic	Fifth
**R**^**2**^	0.9937	0.9993	0.9997	0.9999

##### 3.2.1.2 Examining adjusted R^2^ values

The adjusted R^2^ is a kind of R-squared parameter that is tuned for the number of the models' parameters with respect to the number of designated points. It is a measure of variation around the mean value. This parameter is presented in Eq. (9).

(9) Adj.R2=1−[(SSresidualdfresidual)(SSresidual+SSmodeldfresidual+dfmodel)]=1−[(SSresidualdfresidual)(SStotal−SScurvature−SSblockdftotal−dfcurvature−dfblock)]

Adjusted R^2^ values for Quadratic, Cubic, Quartic and Fifth order models are presented in Table 2 and are respectively equal to 0.9933, 0.9992, 0.9996 and 0.9999. The maximum Adjusted R^2^ value belongs to the fifth order model, which showcases its higher accuracy. As can be observed in the table, higher-order models have higher adjusted R^2^ values.

**Table 2:** ${R_{adj}}^{2}$for different under review models.

Source	Quadratic	Cubic	Quartic	Fifth
**Adjusted R**^**2**^	0.9933	0.9992	0.9996	0.9999

##### 3.2.1.3 Examining predicted R^2^ values

The predicted R^2^ parameter indicates how well a model predicts responses, and is calculated as explained in Eq. (10).

(10) $$\mathrm{Pr}\;ed.\;R^{2}=1-\left[\frac{PRESS}{SS_{residual}+SS_{\mathrm{mod}\;el}}\right]=1-\left[\frac{PRESS}{SS_{total}-SS_{curvature}-SS_{block}}\right]$$

Table 3 It provides the predicted R^2^ values from which we conclude that the fifth order model is very close to 1 and has the highest value.

**Table 3:** Predicted R^2^ for different examined models.

Source	Quadratic	Cubic	Quartic	Fifth
**Predicted R**^**2**^	0.9928	0.9991	0.9995	0.9998

##### 3.2.1.4 Examining Std. Dev. values

This parameter is the square root of the residual mean square. Lower values indicate higher performance. Table 4 is dedicated to this parameter.

**Table 4:** Std. Dev. for second, third, fourth, and fifth-degree models.

Source	Quadratic	Cubic	Quartic	Fifth
**Std. Dev.**	0.4438	0.1557	0.1056	0.0554

#### 3.2.2 Evaluating accuracy-related plots

**Figure 1:**
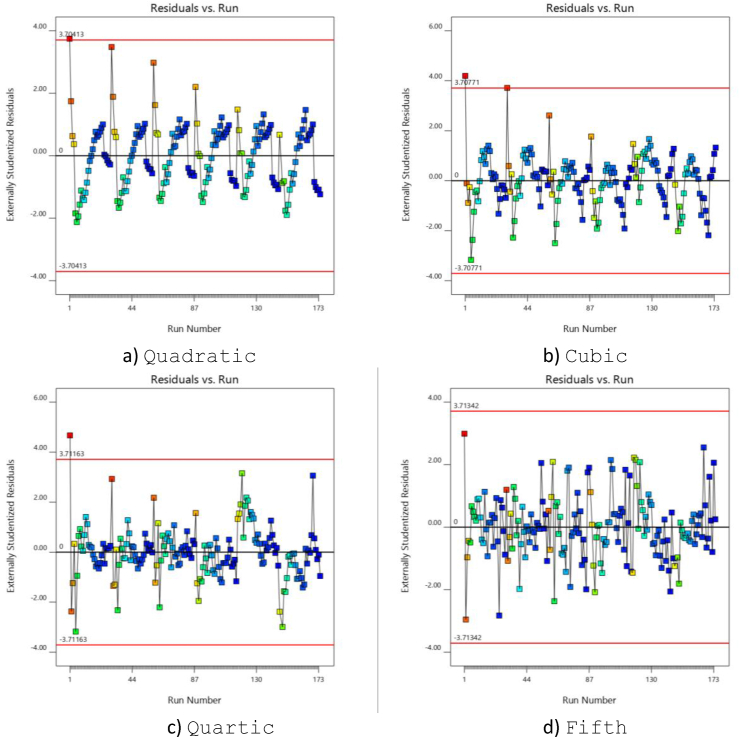
Residual values in terms of Run.

Figure 1 depicts the plot of residual values for different experiments. that the consistency of variance is checked. The randomness of the plot allows for detailed checks. Values should be within a specified range for more accurate modelling. The high amount of variance in this plot indicates that a transfer function must be used. The higher accuracy of the fifth-order model is visible in Figure 1, as the data are well within the range specified with the red line. The plots regarding the second, third and fourth order models show less accuracy than the fifth order model in this respect. Dividing the residual values by their standard deviation obtains the studentized residual values. The calculated standard deviations are observation excluded and therefore, in some cases like Figure 1 (a–d), the term “externally studentized residuals” Is used instead of “studentized residuals”. Studentized residuals compare the observed values of the target and the prediction in the regression condition, with a variety of prediction values.

**Figure 2:**
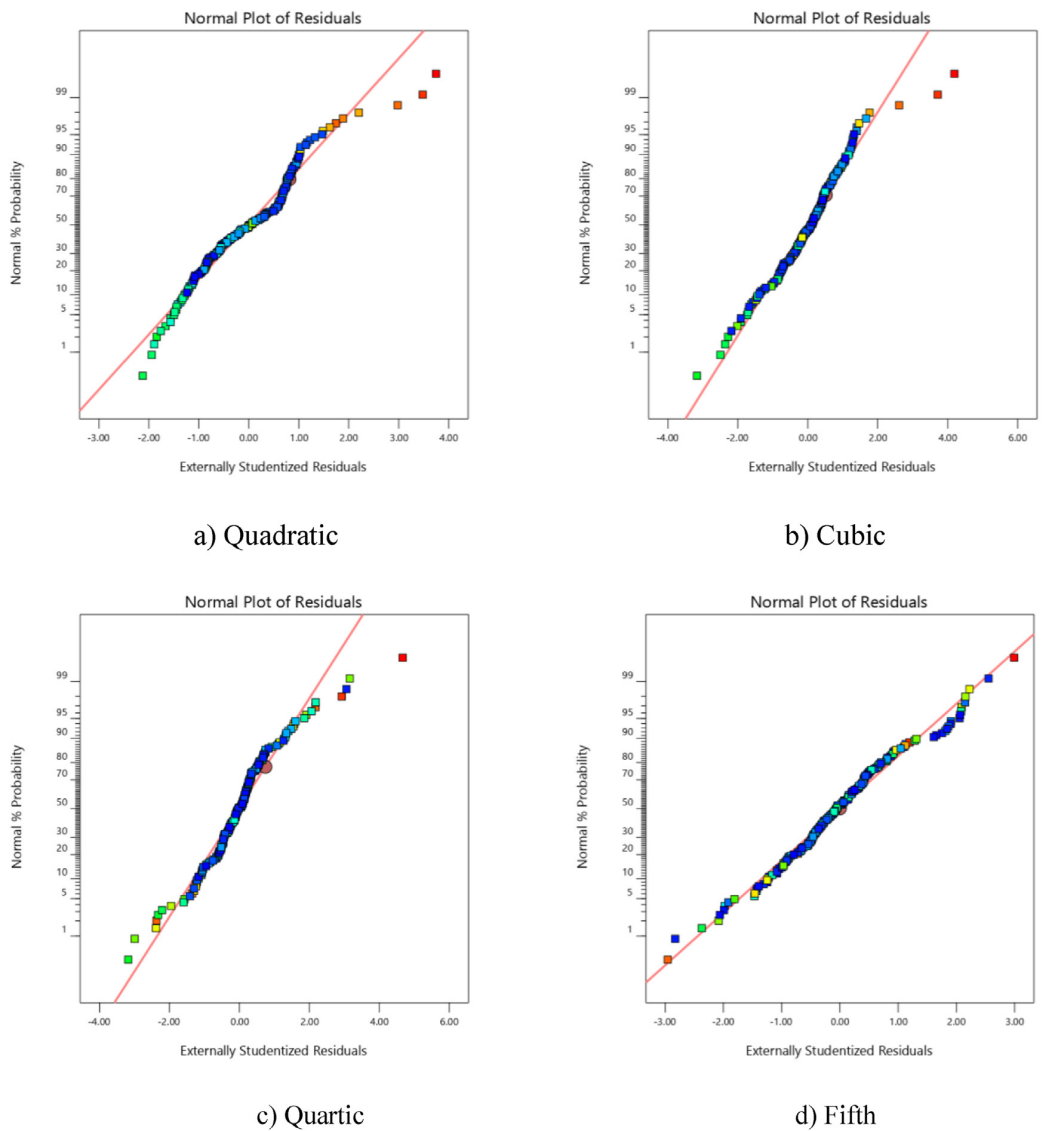
Normal distribution curve in terms of residual values.

Figure 2(a–d) depicts the normal probability graph of different models. These graphs indicate whether the residuals are normally distributed and are linear. Some scattering is expected even with typical data. If the data forms an s-shaped curve, transfer functions must be used. As Figure 2(a–d) indicates, the fifth-order model is mostly linear and has minimal deviation, but significant deviation is observed for the second-order model. To evaluate how normally distributed a small dataset is, normal probability plots are used. Testing the normality of the curve can be performed by plotting the frequency distribution or histograms. To be more specific, a normal probability plot is a Q-Q plot. The plot of the residuals displays the residual values when they are normally distributed, in terms of the expected values.

**Figure 3:**
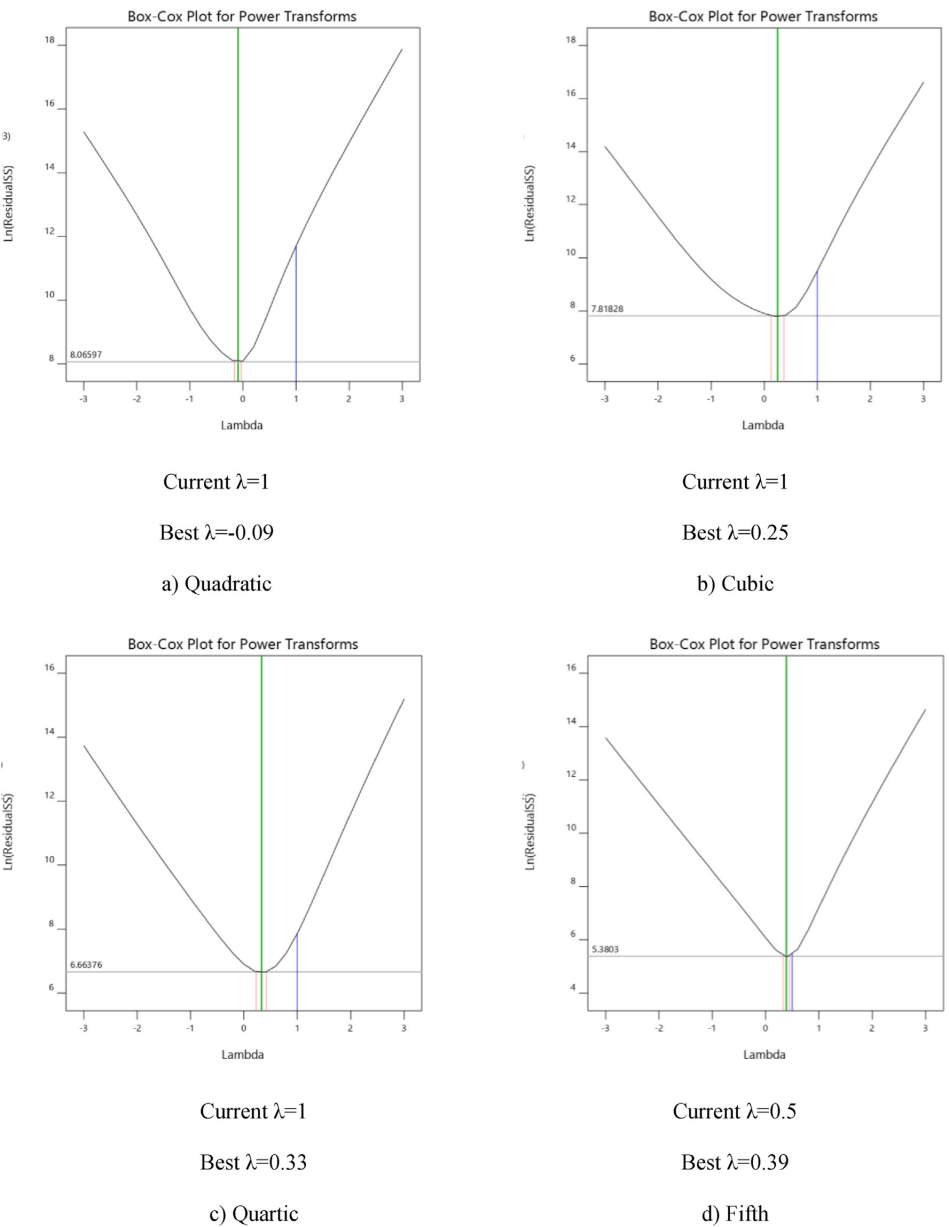
Box-Cox diagrams for determining Lambda values.

In Box-Cox plot analysis, a lambda value of 1 indicates a good fit with the original data. The purpose of using Box-Cox graphs is to convert the data distribution to normal distribution. For example, a lambda value equal to 0.5, indicates that by calculating the square root of the data, they can be the normally distributed. Box-Cox plots of the four models are presented in Figure 3(a–d). These plots provide instructions for choosing the correct transfer function. The optimal transfer function is suggested based on the best lambda value, which is located at the lowest point of the curve. If the 95% confidence interval surrounding this lambda includes 1, The software does not recommend any transformation. As it is displayed in Figure 3(a–d), the plot for the fifth order model showcases appropriate behaviour, and the lambda line is mostly located at the bottom of the curve.

**Figure 4:**
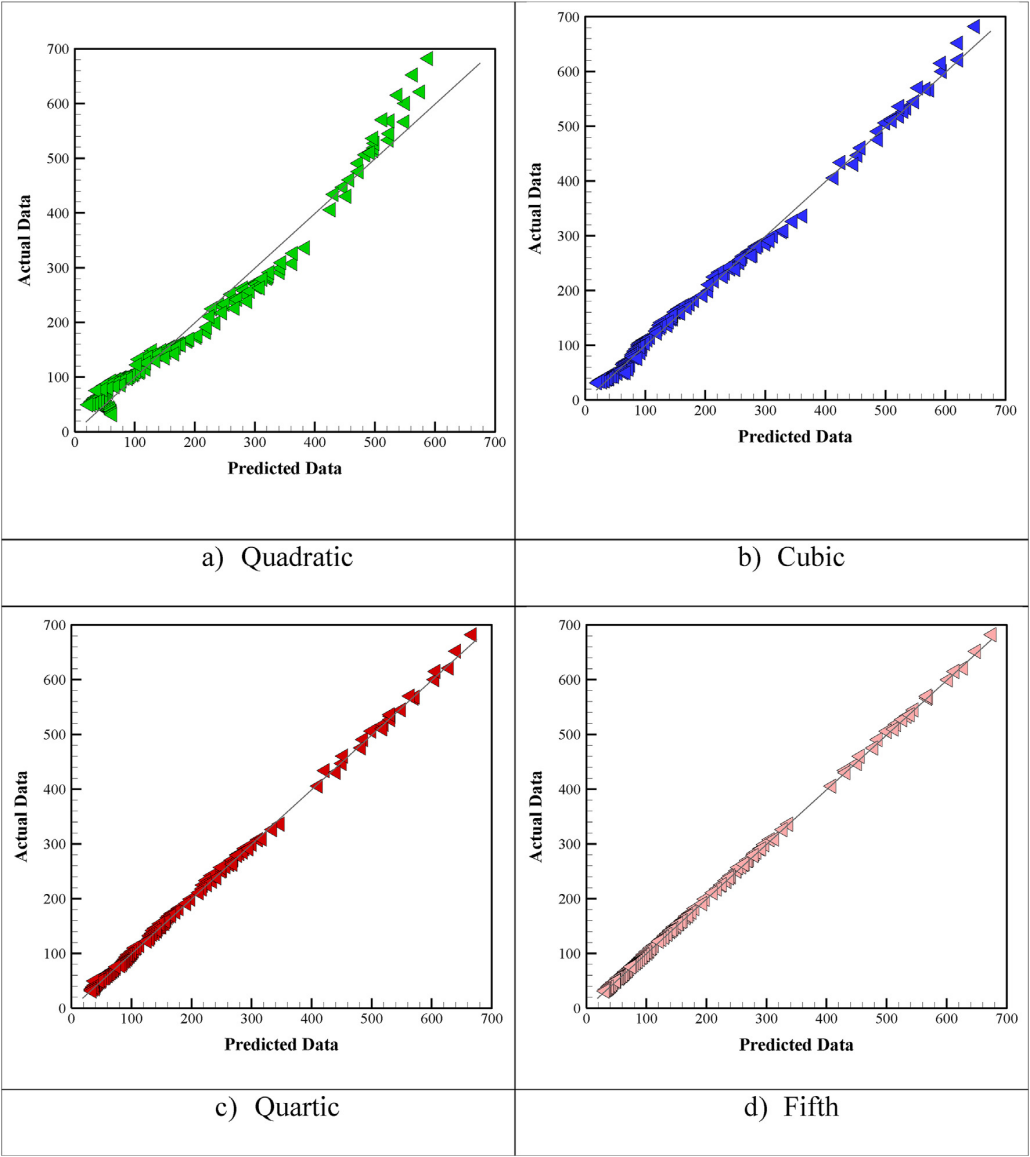
Comparison of predicted and actual values.

Conformity between predicted data and real data can be evaluated using a “Predicted vs. Actual” plot. The 45° bisector of this plot is a measure of conformity. The more data is placed along this line, the more accurate the results are. Figure 4(a–d) displays a plot of predicted response values versus the real response values. Data with noticeable deviation from the true value can be identified using this plot. As is visible in the figure, higher-order models are more accurate in such a way that the fifth-order model is well placed on the bisector line.

According to the presented statistical parameters and plots, it turns out that the fifth order model has the highest accuracy and therefore it is chosen to model MWCNT (40%)/TiO_2_ (60%)/10W40 HNF viscosity. Variance analysis of the selected model is presented in Table 5. As it is visible, the p-values are smaller than 0.05, which Is an indication of the influence of the terms displayed in the table, and their significance in the model.

**Table 5:** ANOVA of the selected model.

Source	Sum of Squares	df	Mean Square	F-value	p-value	
**Model**	5076.62	23	220.72	71,287.44	<0.0001	significant
A-T	202.29	1	202.29	65,335.17	<0.0001	
B-SVF	1.55	1	1.55	501.05	<0.0001	
C-SR	1.68	1	1.68	543.80	<0.0001	
AB	0.7833	1	0.7833	252.99	<0.0001	
AC	0.0612	1	0.0612	19.77	<0.0001	
A^2^	8.30	1	8.30	2679.52	<0.0001	
B^2^	0.3182	1	0.3182	102.77	<0.0001	
C^2^	0.0856	1	0.0856	27.63	<0.0001	
A^2^B	0.1586	1	0.1586	51.22	<0.0001	
A³	2.00	1	2.00	644.50	<0.0001	
B³	0.7641	1	0.7641	246.78	<0.0001	
C³	0.0244	1	0.0244	7.88	0.0057	
ABC^2^	0.0595	1	0.0595	19.22	<0.0001	
B^2^C^2^	0.0242	1	0.0242	7.81	0.0059	
A³B	0.0438	1	0.0438	14.15	0.0002	
AC³	0.9199	1	0.9199	297.12	<0.0001	
A^4^	0.3219	1	0.3219	103.97	<0.0001	
B^4^	0.1355	1	0.1355	43.76	<0.0001	
A³BC	0.1178	1	0.1178	38.04	<0.0001	
A^4^C	0.3130	1	0.3130	101.09	<0.0001	
AB^4^	0.1865	1	0.1865	60.24	<0.0001	
B^5^	0.8539	1	0.8539	275.80	<0.0001	
C^5^	0.2469	1	0.2469	79.73	<0.0001	
**Residual**	0.4613	149	0.0031			
**Cor Total**	5077.09	172				

### 3.3 The trend of viscosity alterations in the selected model

**Figure 5:**
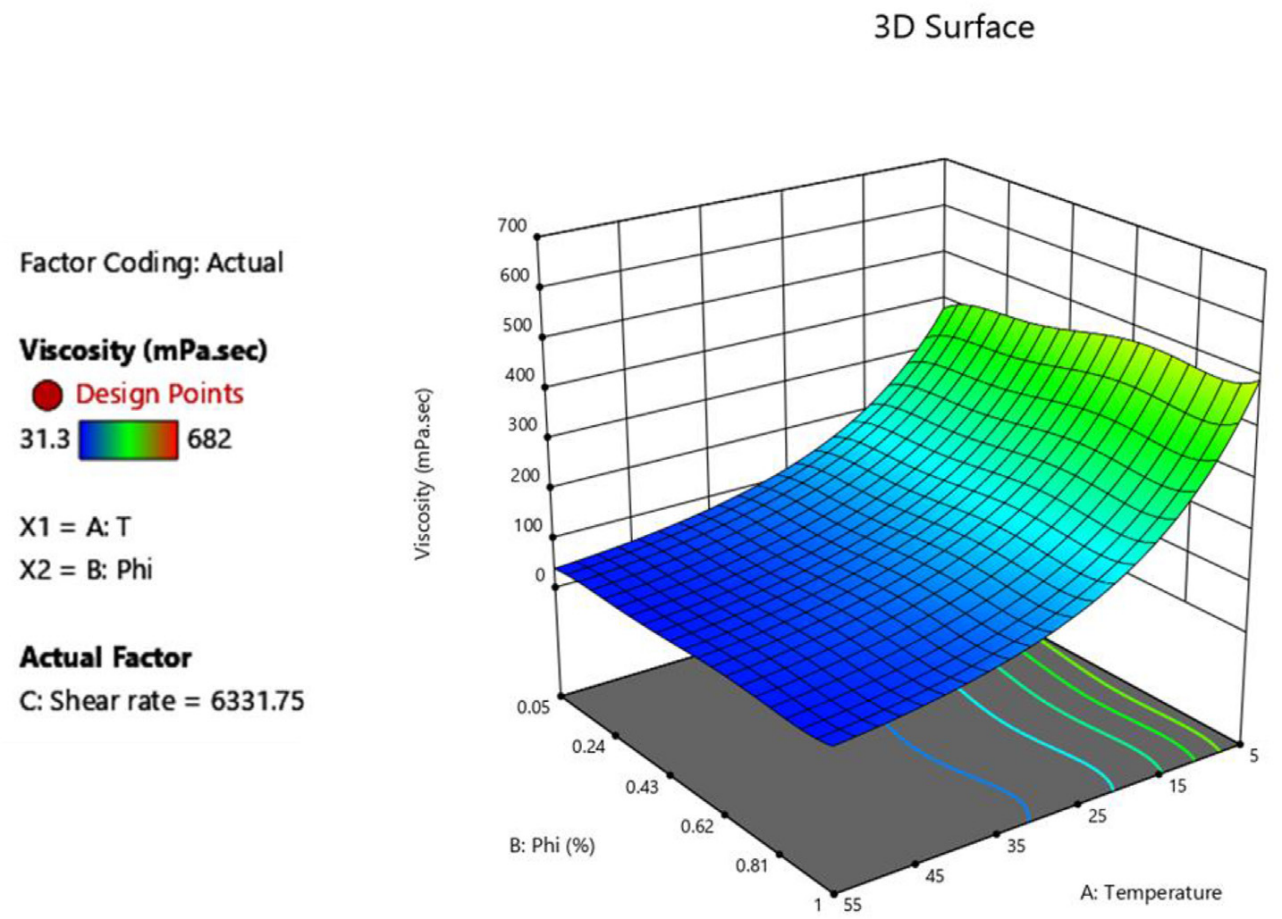
Dynamic viscosity of HNF as a function of T and SVF.

**Figure 6:**
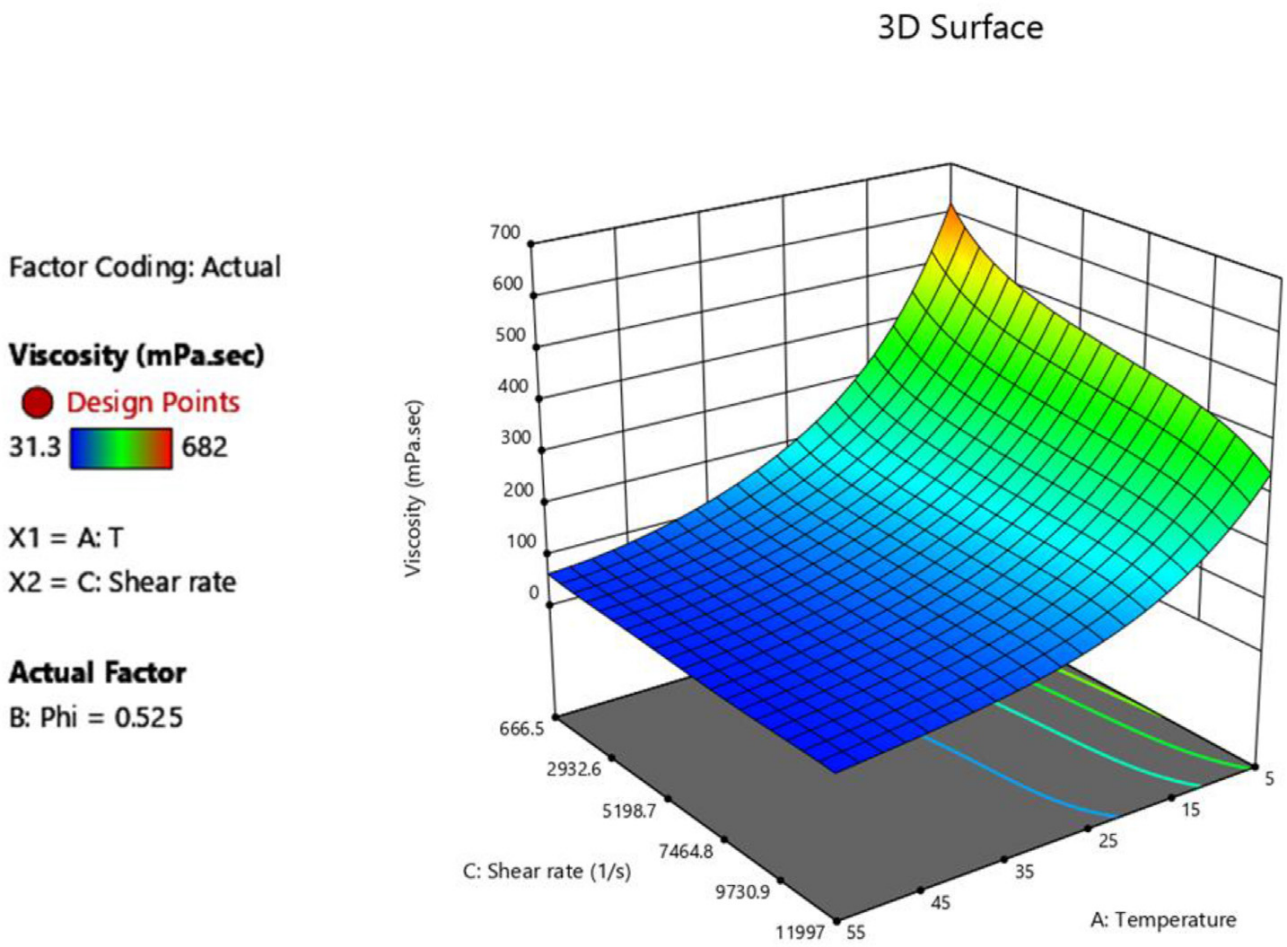
Dynamic viscosity of HNF as a function of T and SR.

A fifth-order model of the viscosity of MWCNT (40%)/TiO_2_ (60%)/10W40 HNF was created using experimental data. This model can be used to calculate viscosity and evaluate its trend. Figures 5 and 6 depict the trend of viscosity alterations. Figure 5 shows changes in µ with temperature and SVF. As it is visible, temperature increments decreased the viscosity. In fact, with the increase in temperature, we will have an increase in the distance between nano particles and base fluid molecules, intermolecular forces are weakened and the layers of the nanofluid can easily slide on each other, therefore the viscosity decreases. Figure 6 displays the trend of µ alterations with respect to T and SR, and increments inSR have had minimal effects on viscosity. Therefore, the SR has caused minimal alterations in the distance between the particles and Van der Waals forces, and has caused negligible changes in the viscosity.

### 3.4 The most effective parameter on the selected model

**Figure 7:**
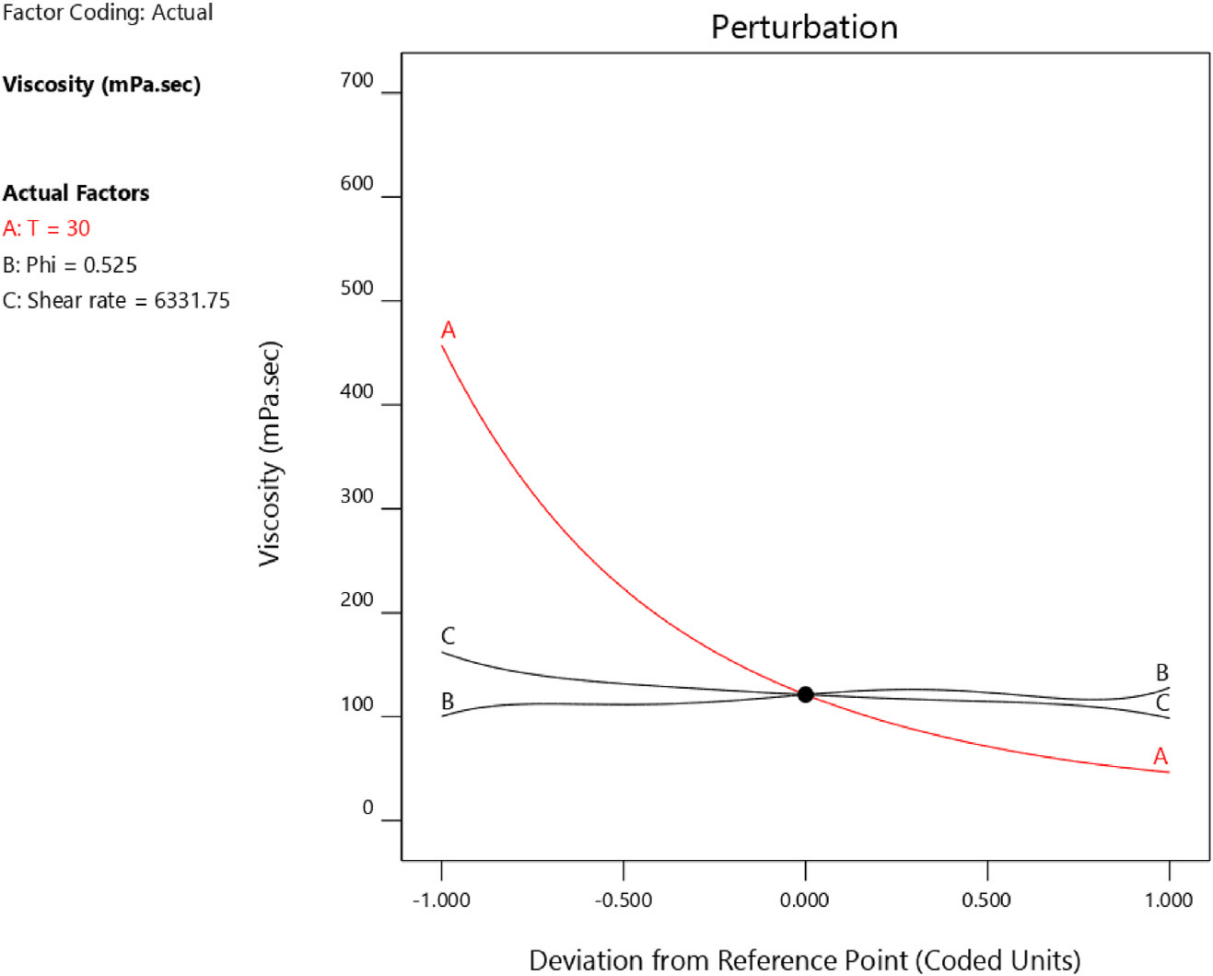
Perturbation curve for viscosity parameters.

Parameters that affect the µ of MWCNT (40%)/TiO_2_ (60%)/10W40 HNF include: T, SVF and SR. In this section, the effective parameter on µ is determined. Using the perturbation plot, the most and least effective parameters can be identified. Figure 7 displays the perturbation plot of the MWCNT (40%)/TiO_2_ (60%)/10W40 HNF. Evidently, temperature is the most effective and SR is the least effective parameter on viscosity. Temperature increments affect both the intermolecular forces and the mobility of the nanoparticles, and as is visible in Figure 7, in the interval of [+1 and −1], viscosity has changed by approximately 400 mPa.sec. Changes in SVF and SR result in minimal effect on viscosity.

### 3.5 Optimizing the viscosity of the nanofluid

Using nanofluids effectively can help save time and resources. The investigated nanofluid consists of MWCNT (40%)/TiO_2_ (60%), and 10W40 oil is the base fluid. This oil has many applications in multiple industries and also in automobiles. It is a practical oil that without adding many nanoparticles, can show a low viscosity at low temperatures. For these reasons, optimization of MWCNT (40%)-TiO_2_ (60%)/10W40 nanofluid is discussed in this section. The specifics of the parameters are presented in Table 6.

**Table 6:** Parameters regarding optimization of dynamic viscosity.

Name	Goal	Lower Limit	Upper Limit	Lower Weight	Upper Weight	Importance
A:T	minimize	5	55	1	1	3
B: SVF	minimize	0.05	1	1	1	3
C:SR	is in range	666.5	11,997	1	1	3
Viscosity	minimize	31.3	682	1	1	3

**Figure 8:**
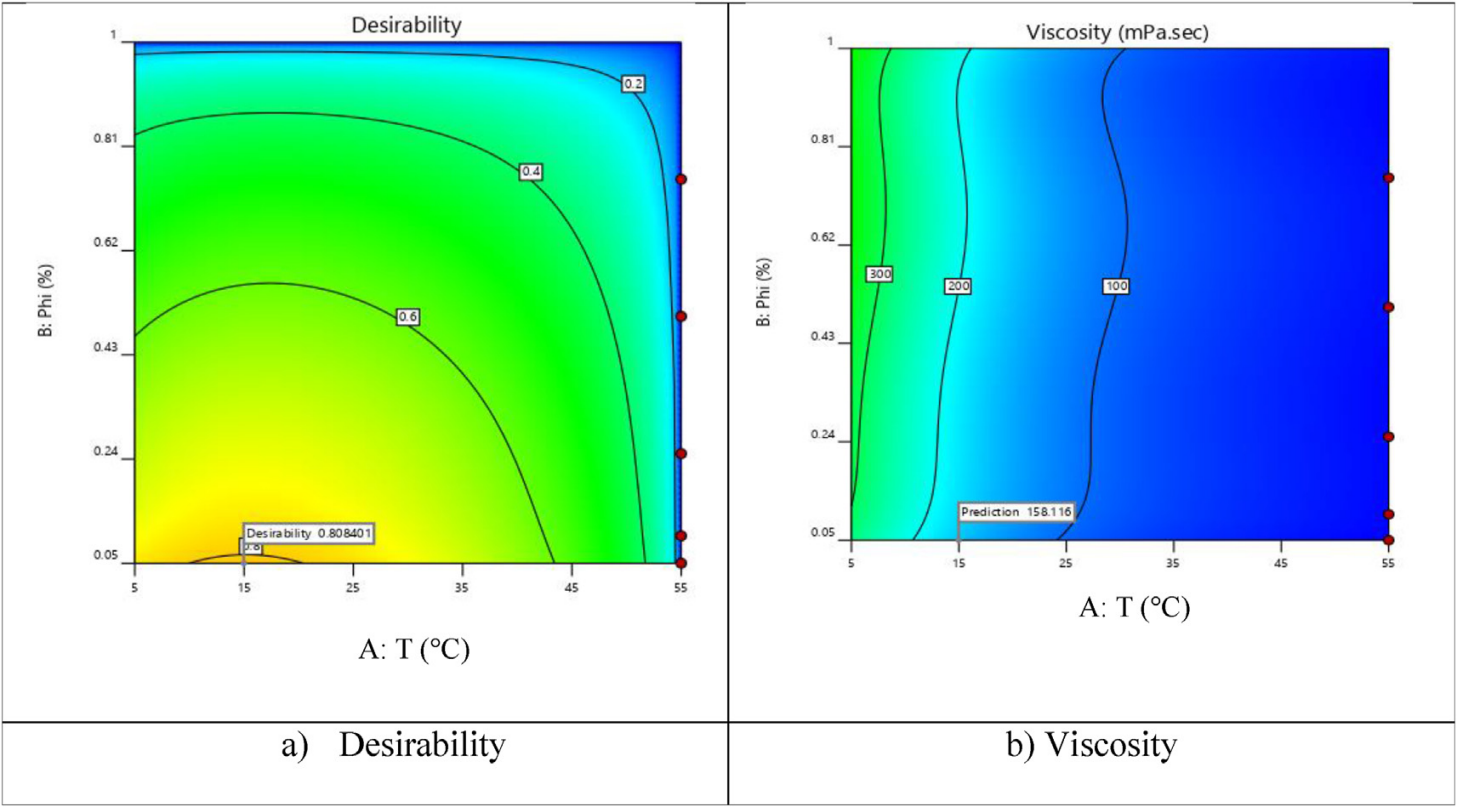
Optimal dynamic viscosity at different SVFs

The optimal value of viscosity for the nanofluid is presented in Figure 8(a, b). As it is visible, optimal viscosity is 158.1 mPa.s at SVF = 0.050%, SR = 11996 s^−^^1^ and T = 14.97 °C. The utility value of this parameter is 0.808.

## 4 Conclusion

Dynamic viscosity of a 10W40 oil-based MWCNT (40%)/TiO_2_ (60%) HNF was investigated in this research. Experimental data were obtained using a viscometer under different conditions and were used to model the characteristics of the fluid’s viscosity. Using modelling methods such as RSM saves time and cuts down costs. Response Surface Methodology was successfully deployed in this research and provided equations to accurately predict the viscosity of the nanofluid. Four equations were presented by the RSM to calculate viscosity, which included quadratic, cubic, quartic and fifth-order equations. The presented equations were based on independent parameters such as T, SVF and SR. Statistical parameters and plots proved the superiority of the fifth-order model compared to the others. R-squared, adjusted R^2^, predicted R^2^ and Std. Dev parameters of the fifth order model were equal to 0.9999, 0.9999, 0.9998 and 0.0554 respectively, this indicates the accuracy of the model. The residual plot, the normal probability plot, the Box-Cox plot and the predicted vs. actual plot also showed that this model is more accurate than the other models, and is well capable of predicting the viscosity of the nanofluid. The effects of different parameters including temperature, SVF andSR on viscosity of HNF were investigated using perturbation plots and temperature was the most influential parameter on the viscosity of the nanofluid, in a way that at a T of 30 °C, SVF = 0.525% and SR = 6331.75 (1/s), alteration of viscosity from the reference point in the [+1 and −1] interval was more than 90%. This noticeable effect is due to the increased mobility of the particles, weakened intermolecular forces and easier sliding of the nanofluids layers on each other, as the temperature rises. A optimal nanofluid for cold seasons is one that has lower viscosity at low temperatures, so proper lubrication is ensured. For the selected model, at SVF = 0.050%, SR = 11,996 (1/s) and T = 14.97 °C, the optimal viscosity is 158.1 mPa.s. Since the science of nanofluids is still very much new, many opportunities for activity and research are available in the field. HNF consisting of MWCNT-TiO_2_ nanoparticles was investigated in this research, but many more are yet to be explored, and experimental research, modelling and simulations for these nanofluids can be considered for future studies. Comparing different nanofluids and choosing the best one is also another research idea. Using the RSM to model process costs and thermophysical properties such as thermal conductivity, density etc., are also among the subjects that require further research.

## Declarations

### Author contribution statement

Mohammad Hemmat Esfe: Analyzed and interpreted the data; Contributed reagents, materials, analysis tools or data; Wrote the paper.

Sayyid Majid Motallebi: Conceived and designed the experiments; Performed the experiments; Analyzed and interpreted the data; Contributed reagents, materials, analysis tools or data.

Davood Toghraie: Conceived and designed the experiments; Performed the experiments; Contributed reagents, materials, analysis tools or data; Wrote the paper.

### Funding statement

This research did not receive any specific grant from funding agencies in the public, commercial, or not-for-profit sectors.

### Data availability statement

No data was used for the research described in the article.

### Declaration of interest's statement

The authors declare no conflict of interest.

### Additional information

No additional information is available for this paper.
